# Observations on the Vaccine Inoculation, with an Account of Its Progress in Austria, and in the East

**Published:** 1802-09-01

**Authors:** 

**Affiliations:** Vienna


THE
Medical and Phyfical Journal.

VOL. VIII.] SEPTEMBER 1, 1802. [NO. XLIII.

*Observations on the Vaccine Inoculation, with an Account of its Progress in Austria, and in the East; communicated by* Dr. DE CARRO, *of Vienna.*

I Have read in the Medical and Phyfical Journal feveral articles refpecting Vaccination; I make, however, the fame remark on your various papers on the fubject, that I do on thofe which are publifhed on the Continent; that is, I obferve a tirefome uniformity on thofe points of the doctrine which are already quite clear, and little likely to throw light on thofe which are ftill obfcure. What belongs to the veterinary part of the doctrine, has not met with a due degree of attention. I wait with the utmoft impatience for a work which I have feen announced in one of your news-papers; *Some Observations on the Origin of the Cow-Pox in the Grease of Horses*, *by* Dr. Loy. I never doubted of Dr. Jenner's affertion, but I always wifhed to fee it put beyond all controverfy. The imperfection of the defcription which the Englifh Vaccinators have hitherto given of the difeafe called *the grease*, has been the only caufe of the fcarcity of experiments which have been made out of England on that point. I can affure you that there is not as yet a fingle veterinary phyfician on the Continent, who knows precifely what difeafe of the heels of horfes correfponds with the Englifh word *grease*.

This fubject of the Jennerian doctrine not being attended with any direct advantage, and neither increafing nor diminifhing the income of Vaccinators in general, has been much neglected. But the connection between the Cow-pox and the Rot in fheep begins to make a lively fenfation. The immenfe benefit which proprietors of fheep-farms would draw from fuch a prefervative is fo evident, that the *auri sacra fames* will do what love for the fciences would never have done. The fubject is purfued with ardor in France; we have the refults of fome pretty favourable experiments; and lately feveral Hungarian proprietors have began the Vaccination of their fheep, and propofe to expofe them afterwards to the rot by every imaginable means. One of thefe experiments is conducted upon a large fcale; 6,000 lambs are now undergoing the vaccination.

The vaccination of dogs has not yet been fufficiently attended to, in proportion to the phyfiological importance which is connected with it, if there is any truth in Dr. Jenner's affertion of its being the means of producing the diftemper, and fecuring dogs from its future attack. Dr. J. has advanced his opinion with his ufual modefty and accuracy, and whatever be the refult of his firft fuggeftion, he is in every refpect fheltered from any fort of reproach. I have collected in my fecond edition all that I knew on the fubject, and Dr. Valentin's experiments are truly interefting. Since its publication I have made feveral experiments; and upon the whole, this is my opinion on the vaccination of dogs: Some of them, but very few, are fufceptible of the true vaccine puftule, from which matter can be taken to vaccinate human beings; as to the production of catarrhal fymptoms, I have feen them but once diftinctly; whether the appearance of thofe fymptoms and of fever is neceffary to preferve a dog from the diftemper, I am not able to determine. I have not yet met with any proprietor of dogs who would give me full power to make upon thofe animals all the experiments I have in view. The poffibility of producing a true Cow-pox, and the impoffibility of producing the Small-pox upon a dog, conftitute, however, a point of diffimilitude in the two difeafes, which makes a pretty ftrong argument in favour of thofe who contend that the two difeafes are different.

Some Englifh travellers have affured me that this affertion of Dr. J. has excited the attention of feveral gentlemen. Lord Grantham and Lord Brooke, with whom I fpoke here lately on the fubject, affured me that they had feen before their departure from England, the pack of Lord Craven vaccinated, in order to verify Dr. J's fecond difcovery. If enlightened proprietors affift phyficians in thofe refearches, that interefting fubject will be foon decided.

I am forry to read in your Journal fo few articles upon the hiftorical part of Vaccination in Europe and other parts of the world. The activity which Continental Phyficians put to its propagation is in every refpect truly praife-worthy. Almoft every Government has given its fanction to it. Thofe who have employed experienced Vaccinators have taken excellent fteps; thofe who have applied for advice to old Regal Cenfors, Counfellors, &c. have adopted the worft. If you wifh to be well informed of all that is going on in the world about Vaccination, you muft abfolutely read the *Sud-preustisch Schlesiche*

*Annalen*

*Annalen der Ausrottung Pocken Impfung*, publifhed by Dr. Friefe and Nowack, of Breflaw. The Hiftorian of the Vaccination will there find the beft materials; and I am perfectly fure that you will draw from them a number of articles which will be very welcome to your Englifh Readers.

I have had the pleafure to propagate Vaccination fo far as Bagdad. You can read a fhort account of it in the laft page of my work; and I have received from Mr. Harford Jones, Britifh Refident at the Court of his Highnefs the Pacha of Bagdad, the account of the arrival of the Vaccine matter, under every poffible form, which I had fent to him along with fix copies of my fecond edition. The Vaccination has begun there under the direction of Dr. Short, an Englifh Phyfician, attached to that miffion.

I have fent lately matter to his Eminency Cardinal Confalvi, Secretary of State to his Holinefs Pius VII. Though in the neighbourhood of Naples, where Dr. Marfhall has introduced the Vaccination, it feems that it is not yet adopted at Rome, or that the matter is extinct.

The King of Pruffia is the firft crowned head who has fubmitted his children to the new method. Therefore, you fee that Dr. Marcus Hers's fophifms have not even biaffed the opinion of his Sovereign.

A law fuit of the moft abominable fort is now going on at Berlin, between a banker of that town and Dr. Wolf. That Phyfician was requefted by the banker to vaccinate his children. He complied with the requeft; but inftead of taking vaccine virus, he took variolous matter of the moft confluent kind. Thofe two children died during the inoculation. The parents, aftonifhed to fee fuch fymptoms produced by the *vaccination*, made the ftricteft inquiry, and traced the evil to its origin. A regular charge was brought againft that monfter, but I do not yet know the refult of it. Dr. Wolf, it is faid, is a friend of Dr. M. Hers; they are both of the Jewifh religion; and it is faid, that W. wanted to pay his court to H. by producing fuch fymptoms as would give fome weight to Dr. H's affertion, that *nothing is to be gained by vaccination, and much to be lost by relinquifhing inoculation.* He probably did not expect that death would be the confequence of the inoculation of thofe two children; but whatever be his punifhment, it will never be equal to the turpitude of his conduct.

My friend Dr. Portenfchlag, who is the moft active Vaccinator at Vienna, wifhing to prove by his example that Vaccination can be undertaken in the earlieft period of life, defired me a week ago to vaccinate his new-born child four hours and a half after its birth. The ceremony was performed in presence of the prieſt and a great number of friends to the vaccination, and the child was chriſtened immediately after, by the name of Mary-Elizabeth-Vaccinia. If one ceremony could be made of the vaccination and chriſtening, would not the ſmall-pox be ſoon entirely eradicated? We have on records the caſe of a child vaccinated twenty-two hours after birth, Mary Elgs. Vaccinia Portenſchlag is ſurely the youngeſt which has hitherto been ſubmitted to this beneficial operation.

I have read juſt now the debates of the Houſe of Commons on the reward granted to Dr. Jenner. I confeſs that I do not think the remuneration adequate to the importance of the diſcovery, the liberality with which Dr. J. has communicated it to the world, and the uſual generoſity of the Engliſh Parliament; but I hope that the different ſocieties and corporations of Great Britain will, as they have ſo often done on ſuch occaſions, ſhew ſeparately their gratitude to the greateſt Medical Benefactor that the world can boaſt of.

I ſend to you a copy of my Second Edition, and an ivory lancet of my invention; though inſignificant, apparently, it ſeems to have powerfully promoted the propagation of the Vaccination, as all other metallic lancets contracted ruſt or verd de gris. The wooden ſheath which my turner has made by œconomy, in order to ſave the expence of quills, which he uſed formerly, ſeems to be an important improvement, becauſe ſome experiments have lately proved that the action of light decompoſes the Vaccine virus. At leaſt, the opacity of this ſheath is the only reaſon I can give for the much more frequent ſucceſs of the ivory lancets ſince a wooden one has been adopted.

If I can be of any ſervice to you, Gentlemen, for information on any part of the Continental Vaccination, I beg you will diſpoſe of me.

*Vienna, June* 20, 1802.

